# Resistance to obinutuzumab-induced antibody-dependent cellular cytotoxicity caused by abnormal Fas signaling is overcome by combination therapies

**DOI:** 10.1007/s11033-022-07280-w

**Published:** 2022-02-26

**Authors:** Natsumi Kawasaki, Yoriko Yamashita-Kashima, Takaaki Fujimura, Shigeki Yoshiura, Naoki Harada, Osamu Kondoh, Yasushi Yoshimura

**Affiliations:** Product Research Department, Chugai Pharmaceutical Co., Ltd., 200 Kajiwara, Kamakura, Kanagawa 247-8530 Japan

**Keywords:** Obinutuzumab, Retreatment, Follicular lymphoma, ADCC, Fas

## Abstract

**Background:**

Obinutuzumab, a Type II anti-CD20 antibody, is used to treat follicular lymphoma. A major mode of action of obinutuzumab is antibody-dependent cellular cytotoxicity (ADCC). Knowledge of the mechanisms of resistance to obinutuzumab is important for the development of next-line strategies to follow obinutuzumab-containing therapy, including obinutuzumab retreatment. Unfortunately, the mechanisms by which tumor cells acquire resistance to ADCC are still poorly understood. To address this, we examined the mechanisms of resistance to obinutuzumab-induced ADCC and the combination efficacy of obinutuzumab and clinically available agents in the established resistant cells.

**Methods and results:**

We established cells resistant to obinutuzumab-induced ADCC using the non-Hodgkin lymphoma cell line RL and examined their mechanisms of resistance and the combination efficacy of obinutuzumab and clinically available agents. Comprehensive analysis by RNA sequencing of resistance mechanisms revealed that abnormal Fas signaling decreased sensitivity to ADCC in resistant clones. Combination treatment with prednisolone, a component of CHOP and CVP, was found to enhance ADCC sensitivity of RL cells and resistant clones and to significantly suppress tumor growth in xenograft models. Treatment with prednisolone upregulated expression of CD20 and an apoptosis-inducing protein BIM, which might augment perforin/granzyme B-mediated cell death. Furthermore, pretreatment of the effector cells with bendamustine enhanced ADCC activity, and treatment with obinutuzumab plus bendamustine showed significant antitumor efficacy in xenograft models. It was speculated that bendamustine upregulates ADCC activity by potentiating granules-mediated cell killing.

**Conclusions:**

Our study revealed a novel mechanism underlying obinutuzumab-induced ADCC resistance and indicated that ADCC resistance could be overcome by combining obinutuzumab with prednisolone or bendamustine. This study provides a scientific rationale for obinutuzumab-retreatment in combination with clinically available chemotherapeutic agents for obinutuzumab resistant follicular lymphoma.

**Supplementary Information:**

The online version contains supplementary material available at 10.1007/s11033-022-07280-w.

## Introduction

Follicular lymphoma (FL) is one of the most common types of indolent non-Hodgkin lymphoma, and its incidence is gradually increasing [1, 2]. Although outcomes for patients with FL have improved over recent decades with rituximab plus chemotherapy [3], most patients relapse and usually cannot be cured. Therefore, there are great unmet needs to develop novel therapeutic strategies and optimize existing therapies.

Obinutuzumab is a glycoengineered type II anti-CD20 monoclonal antibody [4]. In the randomized phase II GAUSS study, obinutuzumab induced a higher overall response rate compared to rituximab [5]. The randomized phase III GADOLIN [6] and GALLIUM [7] trials showed the efficacy of obinutuzumab plus chemotherapy. The GADOLIN trial compared obinutuzumab plus bendamustine with bendamustine alone in patients who did not respond to rituximab. The GALLIUM trial investigated the efficacy of obinutuzumab combined with CHOP (cyclophosphamide, doxorubicin, vincristine, and prednisone), CVP (cyclophosphamide, vincristine, and prednisone), or bendamustine, compared with rituximab-based chemotherapy in previously untreated FL patients. Based on these results, obinutuzumab was approved for previously untreated or relapsed/refractory FL. In the clinical setting, retreatment of FL patients with rituximab has been reported to be effective for relapsed cases [8]. However, owing to the lengthy prognosis of FL and the insufficiently long clinical experience with obinutuzumab as compared to rituximab, there is still no evidence on the effectiveness of retreatment with obinutuzumab in patients who relapse after initial obinutuzumab treatment. This is an important issue to be resolved in the search for next-line therapies.

One of obinutuzumab’s major modes of action involves antibody-dependent cellular cytotoxicity (ADCC). ADCC is a killing process of target cells largely mediated by effector natural killer (NK) cells [9], and enhancement of ADCC is a critical approach in monoclonal antibody-mediated cancer therapy. Through defucosylation in its Fc portion, obinutuzumab has enhanced binding affinity to Fcγ receptor III, resulting in induction of ADCC greater than that of rituximab [4, 10]. The mechanisms involved in this process are including the degranulation of lytic granules, perforin and granzymes, and death receptor signaling such as the FasL–Fas cascade [11, 12]. Both granzymes and Fas signaling initiate apoptosis through multiple pathways [13]. It is important to understand the mechanisms of resistance to obinutuzumab in order to develop next-line strategies; however, relatively little is known about the mechanisms by which tumor cells acquire resistance to ADCC, and previous studies have uncovered only a few candidate genes associated with ADCC resistance (e.g., target antigens, XIAP, and target cell adhesion related genes) [14–16].

In this study, we established cells resistant to obinutuzumab-induced ADCC and investigated their mechanisms of ADCC resistance as well as the effectiveness of prednisolone, a component of CHOP/CVP, or bendamustine to overcome resistance. As a result, our current study provides novel insights into the molecular mechanisms underlying ADCC resistance and the efficacy of obinutuzumab-retreatment against obinutuzumab resistant FL.

## Methods

### Reagents

Obinutuzumab was provided by F. Hoffmann-La Roche Ltd. (Basel, Switzerland). Prednisolone for in vitro assay was purchased from FUJIFILM Wako Pure Chemical Corporation (Osaka, Japan). Prednisolone for in vivo assay was purchased from Shionogi & Co., Ltd. (Osaka, Japan). Bendamustine HCl was purchased from Selleck Chemicals (Houston, TX, USA).

### Cell lines and cell culture

RL, SU-DHL-2, and SU-DHL-4 cells were obtained from the American Type Culture Collection (ATCC, Manassas, VA, USA), and were maintained in RPMI-1640 (Sigma-Aldrich, St. Louis, MO, USA) supplemented with 10% FBS (Nichirei Biosciences Inc., Tokyo, Japan), 10 mM HEPES (Sigma-Aldrich), 0.45% d-glucose (Sigma-Aldrich), and 1 mM sodium pyruvate (Thermo Fisher Scientific, Waltham, MA, USA). CD16 (158V)/NK92 (NK92) cells were established as previously described [17]. NK92 cells were grown in MEMα (FUJIFILM Wako Pure Chemical Corporation) supplemented with 10% FBS, 10% horse serum (Thermo Fisher Scientific), 0.1 mM 2-mercaptoethanol (Thermo Fisher Scientific), 0.02 mM folic acid (Sigma-Aldrich), 0.2 mM myo-inositol (Sigma-Aldrich) and 0.02 μg/mL recombinant human IL-2 (PeproTech, Cranbury, NJ, USA). Farage, DB, HT, NU-DUL-1, SU-DHL-5, SU-DHL-8, and SU-DHL-10 cells were purchased from ATCC and maintained in RPMI-1640 ATCC modification (Thermo Fisher Scientific) with 10% FBS. STR-428 cells were obtained from the Japanese Collection of Research Bioresources Cell Bank (JCRB Cell Bank, Osaka, Japan) and maintained in RPMI-1640 ATCC modification with 10% FBS. The SU-DHL-6 cells from ATCC were obtained from F. Hoffmann-La Roche, under research agreement, and maintained in RPMI-1640 supplemented with 10% FBS, 10 mM HEPES, 0.45% d-glucose, and 1 mM sodium pyruvate. RC-K8 cells were obtained from JCRB Cell Bank and cultured in RPMI-1640 ATCC modification containing 20% FBS. All cells were cultured at 37 °C under 5% CO_2_.

### Establishment of clones resistant to obinutuzumab-induced ADCC

RL cells were pretreated with 300 µg/mL of mutagen *N*-ethyl-*N*-nitrosourea (ENU; Sigma-Aldrich) to establish resistant clones more efficiently by inducing mutagenesis [18], and were then treated with effector NK92 cells at an effector/target (E/T) ratio 20:1 and 0.1 μg/mL of obinutuzumab for 24 h. CD20 positive cells were enriched by magnetic-activated cell sorting using anti-CD20 antibodies (BD Biosciences, San Jose, CA, USA). These ADCC induction and cell sorting procedures were repeated three times, and regrown cells were then single cell cloned.

### ADCC assay

ADCC assay was performed using indicated target cells and NK92 cells as the effector cells. Target cells prelabeled with a cell-permeable fluorescent dye (calcein-AM; FUJIFILM Wako Pure Chemical Corporation) for 1 h were seeded into 96-well plates at 1 × 10^4^ cells/well. For blocking assays, target cells were incubated for 30 min with 10 μg/mL antagonistic ZB4 anti-Fas monoclonal antibodies (GeneTex Inc., Irvine, CA, USA) or 10 μg/mL control IgG1 (Medical & Biological Laboratories Co., Ltd., Nagoya, Japan). Specific lysis was assessed at 4 h after exposure to NK92 cells at an E/T ratio 1:1 in the presence or absence of obinutuzumab. Fluorescence intensity of calcein was measured with a Varioskan LUX Multimode Microplate Reader (Thermo Fisher Scientific). %ADCC was calculated as follows: (experimental release − background)/(maximum lysis − background) × 100. Here, “background” means labeled target cells only and “maximum lysis” means labeled target cells lysed with 1% Triton X-100 (Sigma-Aldrich). The results of ADCC (%) are presented as mean ± SD. All experiments were performed at least twice, and representative results of one experiment are shown.

### In vivo experiments

Female 5-week-old C.B-17/Icr-scid/scidJcl (SCID) mice were obtained from CLEA Japan, Inc. (Tokyo, Japan). Female 5-week-old CB17.Cg-*Prkdc*^*scid*^*Lyst* ^*bg-J*^ /CrlCrlj (SCID Beige) mice were obtained from The Jackson Laboratory Japan (Kanagawa, Japan). Each mouse was inoculated subcutaneously with 5 × 10^6^ tumor cells. Several weeks after tumor inoculation, mice were randomly allocated to control and treatment groups. To evaluate the antitumor activity of the test agents, tumor volume was measured twice a week. The antitumor activity was evaluated from tumor volume as described previously [19]. Human IgG or obinutuzumab was administered intravenously once a week for 3 weeks. Prednisolone or vehicle was administered orally daily from Day 1 to Day 5. Bendamustine or vehicle was intravenously administered on Day 1 and Day 2. Data points represent mean + SD. For assessment of the expression of CD20 in tumor samples, prednisolone or vehicle was administered orally daily from Day 1 to Day 4. All experiments were performed at least twice and the representative data of one experiment are shown.

### Flow cytometry

For in vivo samples, tumors were harvested from SCID mice and digested using Tumor Dissociation Kit human (Miltenyi Biotec B.V. & Co. KG, Bergisch Gladbach, Germany). Cells were labeled with indicated antibodies or isotype control antibodies. Cells were analyzed by BD LSRFortessa X-20 (BD Biosciences) and FlowJo v10 software (Tree Star, Inc., Ashland, OR, USA). Anti-CD20, anti-CD19 antibodies, IgG2bκ isotype control, and IgG1κ isotype control were obtained from BD Biosciences. Anti-CD95 (Fas) antibodies were purchased from BioLegend (San Diego, CA, USA). All experiments were performed at least twice, and representative results are presented as MFI (mean fluorescence intensity).

### Caspase-3/7 assay

Caspase-3/7 activity was measured using a Caspase-Glo 3/7 Assay Kit (Promega Corporation, Madison, WI, USA) in accordance with the manufacturer’s instructions. Caspase-3/7 assay was performed 4 h after treatment with or without agonistic CH-11 anti-Fas antibodies (Medical & Biological Laboratories) and the activity was measured with a Varioskan LUX Multimode Microplate Reader. The results are presented as mean ± SD. All experiments were performed at least twice, and representative results are presented.

### RNA interference

siRNA pools were obtained from Horizon Discovery Group (Cambridge, UK). The following ON-TARGETplus siRNA pools were used: non-targeting control (siNC): D-001810-10-05 and siBCL2L11 (siBIM): L-004383-00-0005. siRNAs were transfected into cells by electroporation using NEPA21 TypeII (Nepa Gene Co., Ltd., Chiba, Japan).

### CD107a assay

Target cells were incubated with effector NK92 cells at an E/T ratio 1:1 for 4 h in the presence of anti-CD107a antibodies or IgG1κ isotype control (BD Biosciences). For the last 3 h, cells were treated with BD Golgi Stop (BD Biosciences). After washing, cells were stained with anti-CD56 antibodies (BioLegend) or IgG1κ isotype control (BD Biosciences). Fluorescence was immediately measured with a BD LSRFortessa X-20. The results were analyzed with FlowJo software. The all experiments were performed at least twice, and the representative results are presented as mean ± SD.

### RNA sequencing

Whole-transcriptome RNA sequencing was conducted by Takara Bio Inc. (Shiga, Japan) using a HiSeq 2500 sequencing system (Illumina, Inc., San Diego, CA, USA). Differential expression (RPKM: reads per kilobase of exon per million mapped reads) was evaluated by using the CLC Genomics Workbench (Qiagen, Hilden, Germany). Pathway analysis was performed with Ingenuity Pathway Analysis software (Qiagen).

### Western blotting

Cells were lysed with cell lysis buffer (Cell Signaling Technology, Inc., Danvers, MA, USA) containing protease inhibitor cocktail (Sigma-Aldrich). Total cell lysates were subjected to SDS polyacrylamide gel electrophoresis and transferred to polyvinylidene difluoride membranes by using an iBlot 2 Dry Blotting System (Thermo Fisher Scientific). Immunoblotting was carried out using the following antibodies; anti-caspase-3, anti-caspase-8, anti-cleaved caspase-8, anti-BIM, anti-Fas, and anti-β-actin antibodies (Cell Signaling Technology). Membranes were incubated with horseradish peroxidase-conjugated secondary antibodies (Cell Signaling Technology), and imaging was performed with a ChemiDoc Touch imaging PC system (Bio-Rad Laboratories, Inc., Hercules, CA, USA). All experiments were performed at least twice and the representative data of one experiment are shown.

### Statistical analysis

All statistical analyses were performed in JMP software (SAS Institute Inc., Cary, NC, USA). Student’s *t*-test was used for paired samples. Dunnett’s test was used for multiple comparisons with the control group. Tukey’s HSD test was used for multiple comparisons within groups. For tumor growth experiments, *P*-values were adjusted for the Wilcoxon rank sum test by the Holm–Bonferroni method. *P*-values of less than 0.05 were regarded as statistically significant. Statistical tests used are described in the figure legends.

## Results

### Establishment and characterization of ADCC resistant RL clones

**Fig. 1:**
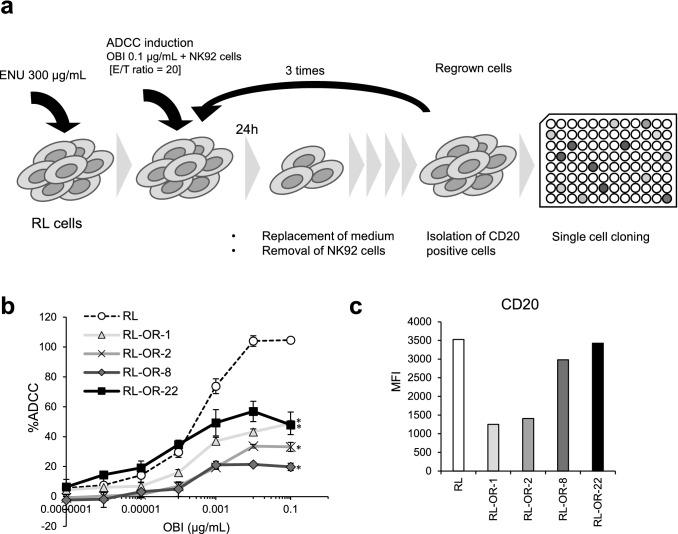
Establishment and characterization of ADCC resistant clones. **a** Schematic diagram of the establishment of clones resistant to obinutuzumab-induced ADCC. **b** ADCC assay was performed with obinutuzumab at indicated concentrations. The results of ADCC (%) are presented as mean values ± SD. *n* = 3, *P < 0.05 by Dunnett's test compared to RL (OBI: 0.1 μg/mL). **c** Surface expression of CD20 on parental cells (RL) and ADCC resistant clones (RL-OR-1, -2, -8, and -22) was measured by flow cytometry and expressed in the graph. *ENU* *N*-ethyl-*N*-nitrosourea; *OBI* obinutuzumab; *E/T ratio* effector/target ratio; *MFI* mean fluorescence intensity

The non-Hodgkin lymphoma cell line RL carries the t(14; 18) chromosomal translocation [20], which is the hallmark of FL [21]. To investigate the efficacy of retreatment with obinutuzumab, we established cells resistant to obinutuzumab-induced ADCC by inducing ADCC with effector NK92 cells and obinutuzumab. To prevent the enrichment of only cells with low CD20 expression, CD20 positive cells were sorted by magnetic-activated cell sorting using anti-CD20 antibodies. The repopulated cells were single cell cloned, and each clone was named RL-OR-1, -2, -8, and -22 (Fig. 1a). In ADCC resistant clones, their sensitivity to ADCC was significantly downregulated compared to parental RL cells (Fig. 1b). To initially characterize the resistant clones, levels of CD20 antigen expression on the cell surface were quantified; flow cytometric analysis demonstrated that resistant clones showed varying levels of surface CD20 expression (Fig. 1c).

### ADCC resistant clones show an abnormal Fas-mediated cell death

We next conducted RNA sequencing to better understand the molecular mechanisms of ADCC resistance, especially in clones with relatively high CD20 expression (RL-OR-8 and -22). Pathway analysis identified several gene sets that were significantly enriched in the resistant clones compared to parental RL cells (Supplementary Table 1). The full list of genes that were significantly enriched in resistant clones compared to parental RL cells is given in Supplementary Table S2. The second-most enriched, after the general category of “molecular mechanisms of cancer”, was “death receptor signaling”. NK cells have been shown to destroy target cells via exocytosis of proteolytic granules, i.e. perforin and granzymes, or engagement of the death receptor system, including the FasL–Fas cascade [11, 12].

**Fig. 2:**
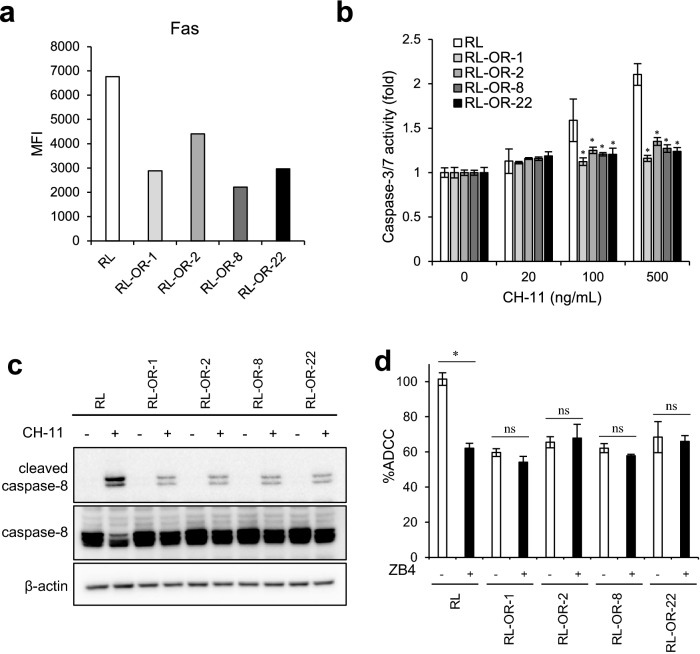
Fas signaling dysregulation inhibits ADCC sensitivity in non-Hodgkin lymphoma RL cells. **a** Surface expression of Fas on parental cells (RL) and ADCC resistant clones (RL-OR-1, -2, -8, and -22) was measured by flow cytometry and expressed in the graph. **b** Cells were treated with indicated concentrations of agonistic anti-Fas antibodies (CH-11) for 4 h before the detection of the caspase-3/7 activity. Data are expressed as the mean ± SD. *n* = 3, *P < 0.05 by Dunnett's test compared to RL for each concentration of CH-11. Differences that were not statistically significant are not illustrated. **c** Cells were treated with or without agonistic anti-Fas antibodies (CH-11, 500 ng/mL) for 4 h. Cell lysates were analyzed by Western blotting. **d** Cells were treated with or without anti-Fas blocking antibodies (ZB4, 10 μg/mL) for 30 min, followed by ADCC assay with obinutuzumab (0.1 μg/mL). The results are presented as mean values ± SD. *n* = 3, *P < 0.05, ns: not significant by Student’s *t*-test for each cell type. *MFI* mean fluorescence intensity

We then examined the expression levels of death receptor Fas and observed that it is downregulated in all four resistant clones (Fig. 2a, and Supplementary Fig. S1a, b). To determine the responsiveness to Fas signaling, we examined the activation of caspase‐3/7 and caspase-8 after stimulation with monoclonal agonistic anti-Fas antibodies (CH-11). In parental RL cells, caspase-3/7 activation was observed in a CH-11-dose-dependent manner. In contrast, CH-11-induced caspase-3/7 activation was reduced in ADCC resistant clones (Fig. 2b and Supplementary Fig. S1c). The reduction of Fas-mediated caspase-8 activation was also observed in resistant clones (Fig. 2c), therefore it was shown that Fas signaling upstream of caspase-8 is deficient in these clones. Then, to explore the contribution of Fas signaling to NK92 cell-mediated ADCC, the effect of blockade with anti-Fas antibodies (ZB4) was assessed by ADCC assay (Fig. 2d). Blocking of Fas signaling significantly inhibited obinutuzumab-induced ADCC activation in parental cells, but had no effect in resistant clones, indicating that suppression of Fas signaling induces decreased sensitivity to ADCC in these four resistant clones.

### Pretreatment of target cells with prednisolone augments ADCC activity

**Fig. 3:**
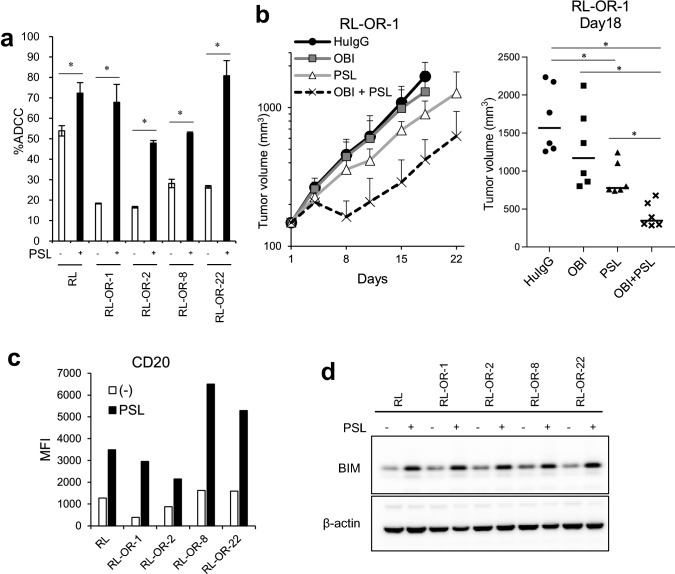
ADCC sensitivity and antitumor efficacy are potentiated by co-treatment with obinutuzumab plus prednisolone. **a** Target cells were pretreated with or without prednisolone (10 μM) for 72 h, and ADCC assay was performed with obinutuzumab (1 ng/mL). The results are presented as mean ± SD. *n* = 3, *P < 0.05 by Student’s *t*-test for each cell type. **b** (Left) SCID mice bearing RL-OR-1 cells were randomly divided into four groups (*n* = 6/group). Control human IgG or obinutuzumab was intravenously administered on Days 1, 8, and 15. Vehicle or prednisolone was orally administered on Day 1 to Day 5. Data points represent mean + SD. (Right) Tumor volumes measured on Day 18, the day when animals reached euthanasia criteria, are displayed. Dots indicate individuals and bars represent median. *P*-values were adjusted for Wilcoxon rank sum test by the Holm–Bonferroni method (*P < 0.05). Differences that were not statistically significant are not illustrated. **c** Cells were treated as in (**a**), and then surface expression of CD20 was measured by flow cytometry and expressed in the graph. **d** Cells were treated as in (**a**), and cell lysates were then collected and analyzed by Western blotting. *HuIgG* human IgG; *OBI* obinutuzumab; *PSL* prednisolone; *MFI* mean fluorescence intensity

To explore the efficacy of obinutuzumab-retreatment, we investigated the efficacy of combinations of clinically available chemotherapeutic agents in FL. Target cells were pretreated with candidate agents and the combination efficacy was assessed by ADCC assay. Among the each single agent of CHOP, pretreatment of cells with prednisolone upregulated ADCC sensitivity in parental cells and ADCC resistant clones (Fig. 3a), suggesting the efficacy of combination therapy with prednisolone. In the xenograft model using RL-OR-1, which shows relatively low CD20 expression, treatment with obinutuzumab (30 mg/kg) in combination with prednisolone (4 mg/kg) significantly increased antitumor activity compared with each single agent on Day 18, the day when animals reached euthanasia criteria in the control and obinutuzumab monotherapy groups (Fig. 3b). The study was conducted up to Day 22 according to the clinical treatment cycle. Pretreatment with prednisolone upregulated the sensitivity to Fas signaling in RL but not in all ADCC resistant clones (Supplementary Fig. S2a), indicating that prednisolone does not directly overcome resistance to obinutuzumab-induced ADCC by activating Fas signaling.

In order to elucidate the mechanisms of this combination treatment, we examined expression of CD20 and BCL2-like 11 (BCL2L11/BIM, an apoptosis-inducing protein), because previous studies reported that they are upregulated upon prednisolone treatment [20, 21]. We found that prednisolone upregulates surface CD20 antigen expression in vitro (Fig. 3c). Prednisolone-induced CD20 upregulation was also observed in the RL-OR-1 xenograft model (Supplementary Fig. S2b). Moreover, the gene expression of BIM was also upregulated from 2 to 72 h after treatment with prednisolone (Fig. 3d, Supplementary Fig. S2c). Knockdown of BIM caused downregulation of ADCC sensitivity in prednisolone-treated parental cells and ADCC resistant clones (Supplementary Fig. S2d, e).

Collectively, our data suggested the possibility that prednisolone shows combination efficacy by upregulating CD20 or BIM expression, which leads to the activation of perforin/granzyme B-mediated cell death rather than Fas signaling.

### Pretreatment of effector cells with bendamustine enhances ADCC activity

**Fig. 4:**
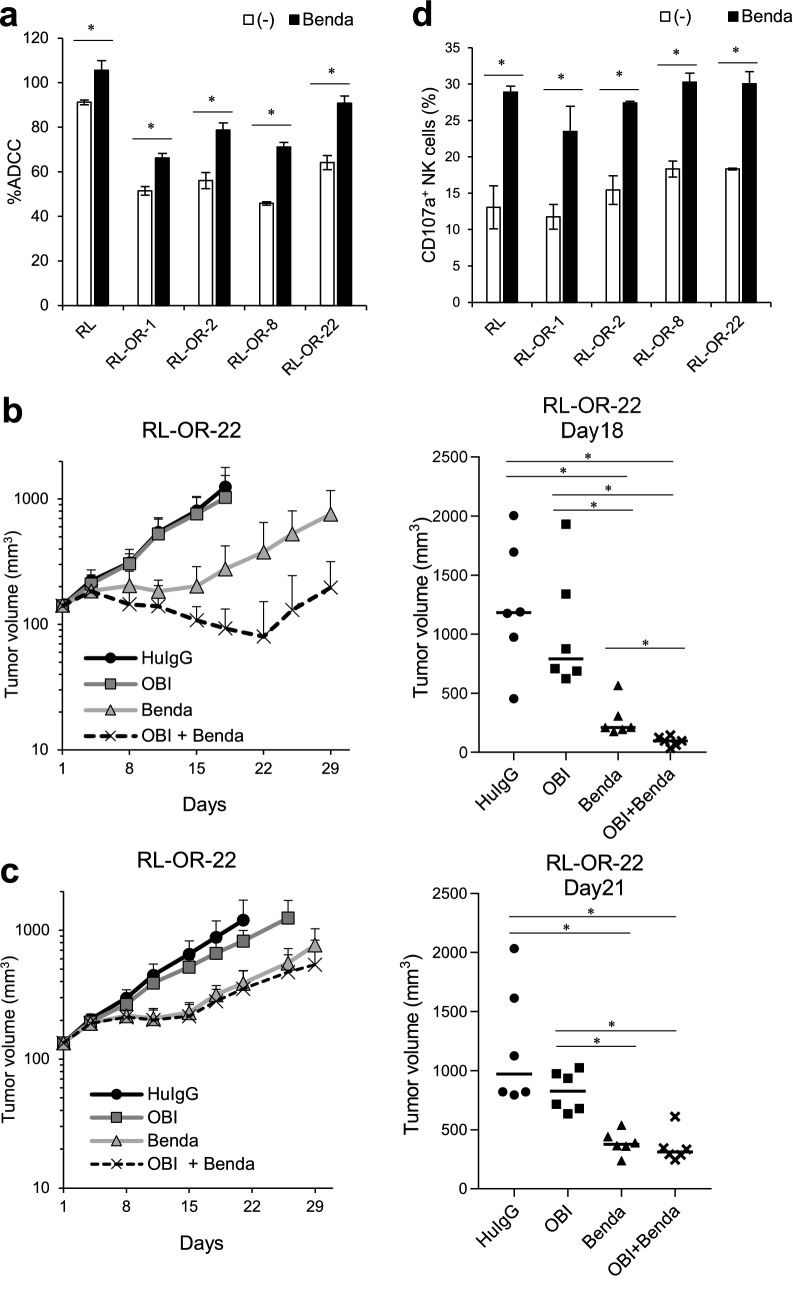
Bendamustine in combination with obinutuzumab potentiates ADCC activity. **a** NK92 cells were pretreated with or without bendamustine (2 μM) for 72 h, and ADCC assay was performed with obinutuzumab (0.1 μg/mL). The results are presented as mean values ± SD. *n* = 3, *P < 0.05 by Student’s *t*-test compared in each cell. **b** (Left) SCID mice bearing RL-OR-22 cells were randomly divided into four groups (*n* = 6/group). Control human IgG or obinutuzumab was intravenously administered on Days 1, 8, and 15. Vehicle or bendamustine was intravenously administered on Day 1 and Day 2. Data represent mean + SD. (Right) Tumor volumes measured on Day 18, the day when animals reached euthanasia criteria, are displayed. Dots indicate individuals and bars represent median. *P*-values were adjusted for Wilcoxon rank sum test by the Holm–Bonferroni method (*P < 0.05). Differences that were not statistically significant are not illustrated. **c** (Left) SCID Beige mice bearing RL-OR-22 cells were treated as in (**b**). Data represent mean + SD. (Right) Tumor volumes measured on Day 21, the day when animals reached euthanasia criteria, are displayed. Dots indicate individuals and bars represent median. *P*-values were adjusted for Wilcoxon rank sum test by the Holm–Bonferroni method (*P < 0.05). Differences that were not statistically significant are not illustrated. **d** NK92 cells were treated as in (**a**), and CD107a assay was performed with obinutuzumab (0.1 μg/mL). The percentage of CD107a^+^ cells among CD56^+^ NK cells was investigated by flow cytometry. The results are presented as mean ± SD. *n* = 3, *P < 0.05 by Student’s *t*-test for each cell type. *HuIgG* human IgG; *OBI* obinutuzumab; *Benda* bendamustine

We also examined the efficacy of bendamustine, another potent combination agent. Pretreatment of effector cells with bendamustine resulted in enhanced ADCC in parental cells and ADCC resistant clones (Fig. 4a), suggesting that bendamustine could alter effector cell functions. Additionally, in the RL-OR-22 xenograft SCID mouse model, which shows relatively high CD20 expression, treatment with obinutuzumab (30 mg/kg) plus bendamustine (13.3 mg/kg) significantly suppressed tumor growth compared with each single agent on Day 18, the day when animals reached euthanasia criteria in the control and obinutuzumab monotherapy groups (Fig. 4b). The study was conducted up to Day 29 in accordance with the clinical treatment cycle. We also examined the combination efficacy in SCID Beige mice which lack NK-cell activity. Combination treatment with obinutuzumab (30 mg/kg) and bendamustine (13.3 mg/kg) did not significantly inhibit tumor growth compared to bendamustine alone on Day 21, the day when animals reached euthanasia criteria in the control group (Fig. 4c). These results indicate that NK cell function is necessary for these combination effects.

To investigate whether bendamustine potentiates NK cell function, CD107a/LAMP1 expression, a marker of NK cell degranulation and activation [22], on NK92 cells after exposure to target cells with obinutuzumab was assessed. Pretreatment with bendamustine increased the ratio of CD107a^+^ NK92 cells after exposing to both parental cells and resistant clones with obinutuzumab treatment (Fig. 4c).

Based on these findings, it is suggested that the combination of bendamustine plus obinutuzumab upregulates ADCC activity by enhancing granules-mediated killing of target cells.

### Combination efficacy in another non-Hodgkin lymphoma cell line

**Fig. 5:**
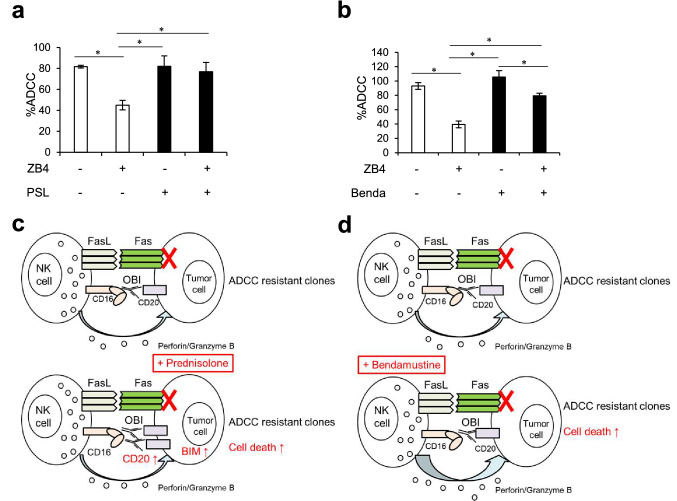
Combination efficacy in Farage cells. **a** Farage cells were pretreated with or without prednisolone (10 μM) for 72 h. Cells were then collected and incubated with or without anti-Fas blocking antibodies (ZB4, 10 μg/mL) for 30 min, followed by ADCC assay with obinutuzumab (0.1 μg/mL). The results are presented as mean ± SD. *n* = 3, *P < 0.05 by Tukey’s HSD test. Differences that were not statistically significant are not illustrated. **b** NK92 cells were pretreated with or without bendamustine (2 μM) for 72 h. Target Farage cells were preincubated with or without anti-Fas blocking antibodies (ZB4, 10 μg/mL) for 30 min, followed by ADCC assay with obinutuzumab (0.1 μg/mL). The results are presented as mean ± SD. *n* = 3, *P < 0.05 by Tukey’s HSD test. Differences that were not statistically significant are not illustrated. **c** Model of combination effect of obinutuzumab plus prednisolone. Pretreatment with prednisolone upregulates CD20 and BIM, and activates perforin and granzymes mediated cell killing. **d** Schematic diagram of bendamustine-mediated ADCC activation. Pretreatment of NK cells with bendamustine enhances NK cell degranulation and activation. *OBI* obinutuzumab; *PSL* prednisolone; *Benda* bendamustine

Finally, to examine the applicability of these models, the expression level of Fas in lymphoma cell lines was evaluated (Supplementary Fig. 3). Both Farage and RL cells showed similar surface expression levels of Fas; therefore, we examined the role of Fas signaling and combination efficacy in Farage cells. We observed consistent results concerning the inhibition of ADCC by blocking of Fas signaling using blocking antibodies (ZB4) in Farage cells, and pretreatment with prednisolone overcame this ADCC inhibition (Fig. 5a). In addition, pretreatment of effector cells with bendamustine resulted in enhanced NK92 cell-induced ADCC in Fas-inhibited Farage cells (Fig. 5b). Together, these results suggest that Fas deficiency could cause obinutuzumab-induced ADCC resistance in several cell types and that combination with prednisolone or bendamustine could overcome these resistances (Fig. 5c, d).

## Discussion

The current study presents a comprehensive analysis of mechanisms of resistance to obinutuzumab-induced ADCC, and to our knowledge, is the first to reveal that abnormalities in Fas signaling can cause ADCC resistance. Fas is a transmembrane death receptor involved in cell death signaling; FasL is predominantly expressed on NK cells and T cells. The activation of Fas leads to recruitment of the cytosolic adaptor molecule FADD and caspase-8 and initiates the apoptotic cascade. We showed here that the expression levels of Fas are reduced and Fas signaling upstream of caspase-8 is deficient in the ADCC resistant RL clones. Because Fas signaling is a common mechanism of action in ADCC, impairment of Fas signaling probably also causes resistance to other ADCC-inducing antibodies other than obinutuzumab. Thus, further investigation would likely identify candidate biomarkers able to predict sensitivity to a variety of ADCC-mediating monoclonal antibodies.

Intriguingly, all of the obinutuzumab-induced ADCC resistant RL clones established here showed Fas signaling deficiency. This implies the importance of Fas signaling in conducting ADCC. In addition, Fas is highly expressed on germinal center B cells, and downregulation of Fas on B cells has been shown to be a mechanism through which they evade immune surveillance during the development of B cell lymphoma [23]. Alterations of Fas or other mutations that impair the FasL–Fas apoptotic pathway have been reported to be prominent in B cell malignancies and associated with poor prognosis [24, 25]. These findings imply that Fas signaling abnormalities are also potential mechanisms underlying relapsed and refractory FL after treatment with obinutuzumab in clinical practice.

Our non-clinical study in the ADCC resistant clones validated the efficacy of readministration of obinutuzumab combined with prednisolone. Prednisolone, which is classified as a glucocorticoid, is commonly used to treat non-Hodgkin lymphoma. Glucocorticoid bound to glucocorticoid receptors translocates into the nucleus, and controls transcription of multiple target genes through glucocorticoid response elements (GREs) [26]. Here we found that the expression of BIM, a member of Bcl-2 family, is upregulated by prednisolone as previously reported [27]. Since it is known that human promoter of BIM gene does not contain a GRE, we hypothesize that a GRE-dependent upstream regulator indirectly controls BIM transcription. Several anticancer drugs has been shown to induce the killing of tumor cells by upregulating BIM expression [28, 29]. Our data showed that knockdown of BIM inhibits ADCC sensitivity in prednisolone-treated parental cells and ADCC resistant clones. Based on these results, it was speculated that prednisolone enhances ADCC sensitivity through upregulating proapoptotic BIM expression.

As has been previously reported with acute lymphoblastic leukemia samples [20], we found that prednisolone upregulates surface expression of CD20, the target antigen of obinutuzumab. It has been found that the expression levels of the target antigens correlate with sensitivity to cetuximab-induced ADCC [30]. Thus, although the direct effect of CD20 upregulation remains to be determined in our resistant cells, it could also contribute to enhancing ADCC sensitivity. Loss or reduction of CD20 expression is known to be one of the mechanisms of resistance to the anti-CD20 monoclonal antibodies [31]. Further studies therefore may show the usefulness of prednisolone for reducing the emergence of such resistance.

Obinutuzumab has various mechanisms of action other than ADCC, including antibody-dependent cellular phagocytosis and direct cell death. We previously demonstrated that co-treatment with obinutuzumab plus prednisolone shows combined effects by enhancing DNA fragmentation and G0/G1 arrest in direct cell death resistant models [32]. Although it is unclear that the effect of BIM or CD20 induction on obinutuzumab to induce direct cell death, combination treatment with obinutuzumab plus prednisolone is expected to address a variety of resistance mechanisms.

Our data also showed the effectiveness of obinutuzumab plus bendamustine, an alkylating agent that shows a unique cytotoxicity [33]. Bendamustine therapy causes prolonged lymphopenia [34], however, the effects of bendamustine on NK cell function at the point of administration remain to be elucidated. Our results showed that pretreatment of NK92 cells with bendamustine enhances degranulation of NK92 cells after exposure to target cells with obinutuzumab. Therefore, treatment with bendamustine may act advantageously with NK cell function under short-term conditions by enhancing perforin/granzyme B-mediated pathway.

## Conclusions

We currently clarify the mechanisms of resistance to obinutuzumab-induced ADCC caused by abnormal Fas signaling and presents the possibility that obinutuzumab in combination with prednisolone or bendamustine could overcome this resistance. Although further investigations are needed to evaluate the clinical efficacy, this is the first report to show the possibility that obinutuzumab-retreatment would be effective against obinutuzumab resistant non-Hodgkin lymphoma.

## Supplementary Information

Below is the link to the electronic supplementary material.

Supplementary file1 (PDF 105 KB)

Supplementary file2 (PDF 164 KB)

Supplementary file3 (PDF 765 KB)

## Data Availability

The datasets used and/or analyzed during the current study are available from the corresponding author on reasonable request.

## Abbreviations

ADCC: Antibody-dependent cellular cytotoxicity
FL: Follicular lymphoma
CHOP: Cyclophosphamide, doxorubicin, vincristine, and prednisone
CVP: Cyclophosphamide, vincristine, and prednisone
NK cells: Natural killer cells
ENU: *N*-ethyl-*N*-nitrosourea
E/T ratio: Effector/target ratio
MFI: Mean fluorescence intensity
GREs: Glucocorticoid response elements
OBI: Obinutuzumab
HuIgG: Human IgG
PSL: Prednisolone
Benda: Bendamustine
