# Supplementary material for: Resistance to obinutuzumab-induced antibody-dependent cellular cytotoxicity caused by abnormal Fas signaling is overcome by combination therapies
Source: Mol Biol Rep. 2022 Feb 26;49(6):4421–33. doi: 10.1007/s11033-022-07280-w (PMC9262784; doi:10.1007/s11033-022-07280-w)
Supplement: Supplementary file 1 — Supplementary file1 (PDF 105 KB) [file 11033_2022_7280_MOESM1_ESM.pdf]

Supplementary Table S1

Top Canonical Pathways	p-value
Molecular Mechanisms of Cancer	9.11E-08
Death Receptor Signaling	2.58E-06
Factors Promoting Cardiogenesis in Vertebrates	3.52E-06
Epithelial Adherens Junction Signaling	8.77E-06
Type I Diabetes Mellitus Signaling	1.06E-05

Gene sets significantly enriched in resistant clones compared to parental RL cells. Gene expression data obtained by RNA sequencing in parental RL cells, and ADCC- resistant clones (RL-OR-8, and -22) were used for pathway analysis.
