# Supplementary material for: Resistance to obinutuzumab-induced antibody-dependent cellular cytotoxicity caused by abnormal Fas signaling is overcome by combination therapies
Source: Mol Biol Rep. 2022 Feb 26;49(6):4421–33. doi: 10.1007/s11033-022-07280-w (PMC9262784; doi:10.1007/s11033-022-07280-w)
Supplement: Supplementary file 2 — Supplementary file2 (PDF 164 KB) [file 11033_2022_7280_MOESM2_ESM.pdf]

Supplementary Table S2

Molecular Mechanisms of Cancer	Death Receptor Signaling	Factors Promoting Cardiogenesis in Vertebrates	Epithelial Adherens Junction Signaling	Type I Diabetes Mellitus Signaling
ADCY9	CASP3	ACVR1	ACVR1	CASP3
APH1B	CASP6	ACVR2A	ACVR2A	CD80
ARHGEF6	CASP7	BMPR1A	AFDN	FAS
ARHGEF11	CASP10	FZD1	CTNNA1	HLA-B
ARHGEF17	FAS	FZD3	CTNND1	HLA-DOA
BBC3	IKBKE	FZD5	FER	HLA-DOB
BCL2L11	MAP3K5	LEF1	HGF	HLA-F
BMPR1A	PARP8	LRP5	JUP	IKBKE
BRAF	PARP9	LRP6	LEF1	IL1B
CASP3	PARP12	PRKCA	MET	IL1RAP
CASP6	PARP15	PRKCZ	NECTIN1	JAK2
CASP7	RIPK1	SMO	NOTCH4	MAP3K5
CASP10	TANK	TCF4	PARD3	PRF1
CDK18	TIPARP	TCF7L1	RALB	RIPK1
CTNNA1	TNF	TGFB3	RRAS2	SOCS1
CTNND1	TNFSF10	TGFBR2	SRC	SOCS5
FAS	TNFSF15	TGFBR3	TCF4	STAT1
FYN			TCF7L1	TNF
FZD1			TGFBR2	
FZD3			TGFBR3	
FZD5			TUBA8	
GAB1			ZYX	
GNAZ				
IRS1				
ITGB1				
JAK2				
LEF1				
LRP5				
LRP6				
MAP3K5				
PAK1				
PLCB2				
PRKAG2				
PRKCA				
PRKCZ				
PTCH1				
RALB				
RASGRF2				
RASGRP1				
RRAS2				
SMAD3				
SMO				
SRC				
TCF4				
TGFB3				
TGFBR2				

Full gene lists in each gene sets, that were significantly enriched in resistant clones compared to parental RL cells, were displayed.
