# Supplementary material for: Resistance to obinutuzumab-induced antibody-dependent cellular cytotoxicity caused by abnormal Fas signaling is overcome by combination therapies
Source: Mol Biol Rep. 2022 Feb 26;49(6):4421–33. doi: 10.1007/s11033-022-07280-w (PMC9262784; doi:10.1007/s11033-022-07280-w)
Supplement: Supplementary file 3 — Supplementary file3 (PDF 765 KB) [file 11033_2022_7280_MOESM3_ESM.pdf]

Fig. S1

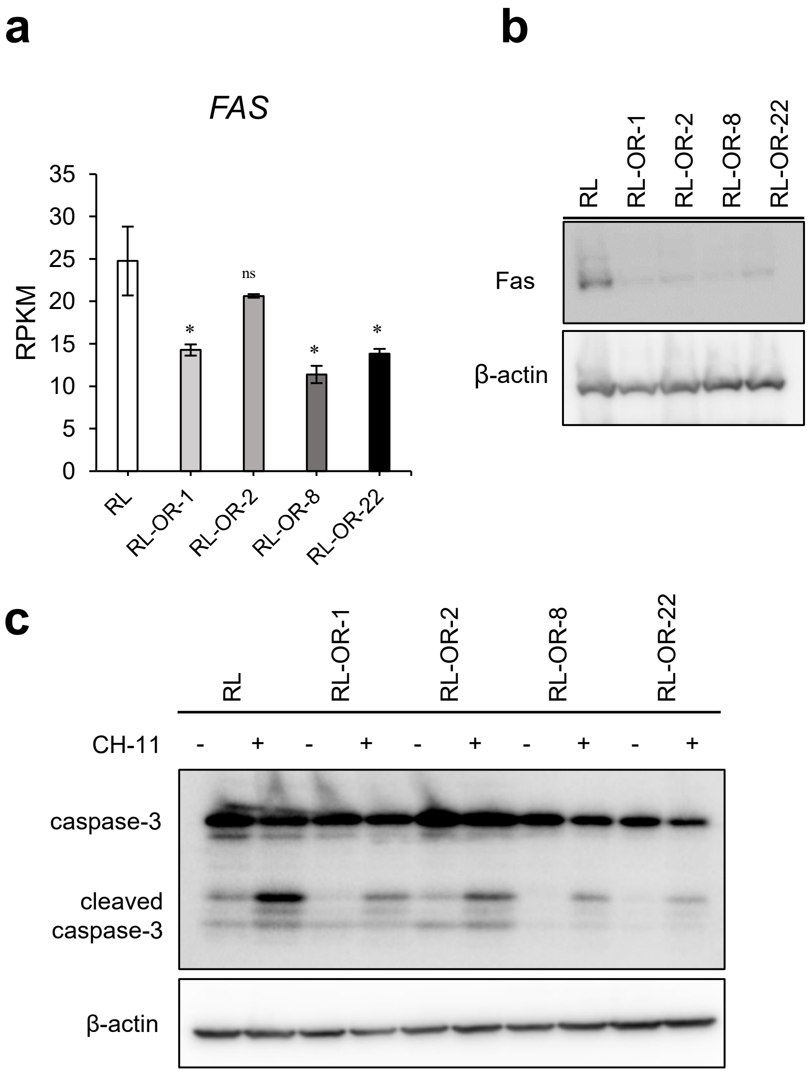

Supplementary Figure S1: Fas signaling is impaired in ADCC resistant clones.
a Gene expression data of *FAS* obtained by RNA sequencing. Data are presented as mean ± SD. $n = 3$, *$P < 0.05$, ns: not significant by Dunnett's test compared to RL. **b** Cells were collected, and cell lysates were analyzed by western blotting. **c** Cells were treated with agonistic anti-Fas antibodies (CH-11, 500 ng/mL) for 4 hours, and cell lysates were analyzed by western blotting

Fig. S2

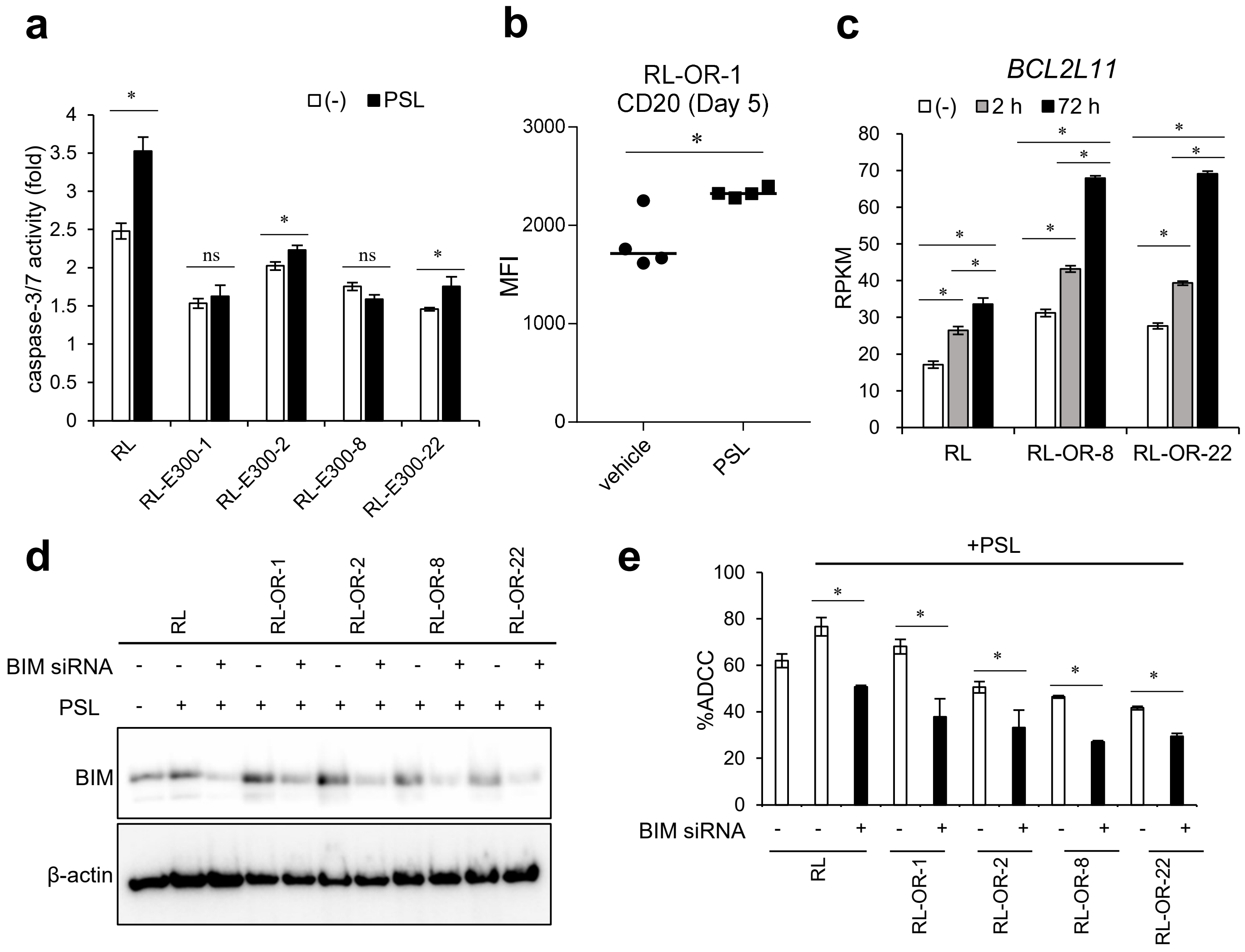

Supplementary Figure S2: Pretreatment with prednisolone upregulates CD20 and BIM expression.
a Cells were treated with or without prednisolone (10 μM) for 72 hours. Cells were then collected and treated with agonistic anti-Fas antibodies (CH-11, 500 ng/mL) for 4 hours before the detection of the caspase-3/7 activity. Data are presented as mean ± SD. n = 3, *$P < 0.05$, ns: not significant by Student's t-test for each cell type. **b** SCID mice bearing RL-OR-1 cells were orally administered with vehicle or prednisolone (4 mg/kg) on Days 1 to 4. Tumor samples were collected on Day 5, and the surface expression of CD20 on CD19 positive cells was measured by flow cytometry. Dots indicate individuals and bars represent median. n = 4, *$P < 0.05$ by Student's t-test. **c** Gene expression data (BCL2L11) obtained by RNA sequencing in cells treated with or without prednisolone (10 μM) for the indicated number of hours. Data are presented as mean ± SD. n = 3, *$P < 0.05$ by Tukey's HSD test for each cell type. **d** Target cells transfected with the indicated siRNAs were treated as in (**a**). Cell lysates were then analyzed by western blotting. PSL: prednisolone. **e** Target cells transfected with the indicated siRNAs were treated as in (**a**), and ADCC assay was performed with obinutuzumab (1 ng/mL). The results are presented as mean ± SD. n = 3, *$P < 0.05$ by Student's t-test for each cell type. PSL: prednisolone; MFI: mean fluorescence intensity

Fig. S3

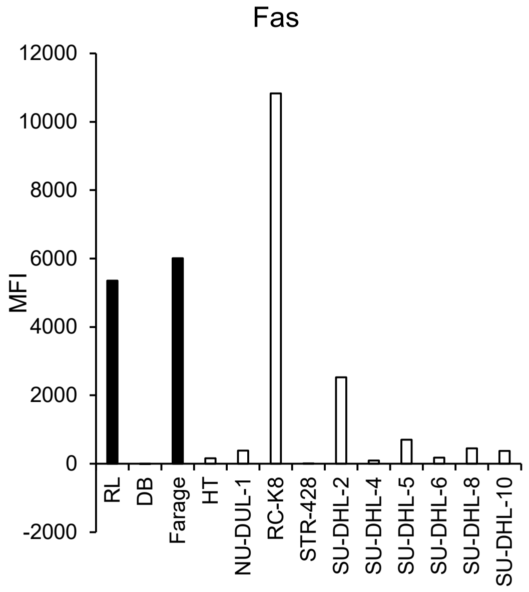

Supplementary Figure S3: Farage and RL cells showed similar surface expression levels of Fas.
a Surface expression of Fas on indicated cell lines was measured by flow cytometry and expressed in the graph. MFI: mean fluorescence intensity
